# DNM3OS regulates GAPDH expression and influences the molecular pathogenesis of Huntington’s disease

**DOI:** 10.1111/jcmm.16838

**Published:** 2021-08-08

**Authors:** Xiaoyu Dong, Shuyan Cong

**Affiliations:** ^1^ Department of Neurology Shengjing Hospital of China Medical University Shenyang China

**Keywords:** DNM3OS, GAPDH, Huntington's disease, long non‐coding RNA, miR‐196b‐5p, therapy

## Abstract

Emerging studies have suggested that dysregulated long non‐coding RNAs (lncRNAs) are associated with the pathogenesis of neurodegenerative diseases (NDD) including Huntington's disease (HD); however, the pathophysiological mechanism by which lncRNA dysregulation participates in HD remains to be elucidated. Here, we aim to analyse the expression of lncRNA‐DNM3OS and identify the possible DNM3OS/miR‐196b‐5p/GAPDH pathway. PC12 cells induced by rat pheochromocytoma expressing HD gene exon 1 fragment with either 23 or 74 polyglutamine repeats fused to the green fluorescent protein (GFP) were cultured. Our results show that GAPDH and DNM3OS were upregulated in HD PC12 cells, downregulation of which lead to inhibition of aggregate formation accompanied by a decreased apoptosis rate and increased relative ROS levels and cell viability. Moreover, upregulated DNM3OS decreased the expression of miR‐196b‐5p by sponging, and GAPDH was a direct target of miR‐196b‐5p, playing an important pathogenic role in the formation of aggregates in the HD cell model. Our study uncovers a novel DNM3OS/miR‐196b‐5p/GAPDH pathway involved in the molecular pathogenesis of HD, which may offer a potential therapeutic strategy for HD.

## 1 INTRODUCTION

Huntington's disease (HD) is an inherited, progressive and fatal neurodegenerative disease (NDD) caused by a CAG trinucleotide expansion in exon 1 of the huntingtin gene (*HTT*).^1^ Even though the exact pathogenesis of HD is still being explored, cell and animal models have demonstrated that amplified poly Q changes the conformation of the mutant Htt (mHtt) protein, and its consequent propensity to self‐aggregate into intracellular inclusion bodies (IBs) may be the key underlying mechanism to pathogenesis.^2^, ^3^

Long non‐coding RNA (LncRNA)‐dynamin 3 opposite strand (DNM3OS) is a lncRNA located on chromosome 1, which is inserted into an independent transcriptional unit in the intron of the dynamin 3 gene.^4^ Previous studies have shown that DNM3OS is involved in a variety of pathophysiological processes and a variety of miRNAs related to HD have a direct regulatory relationship with DNM3OS.^5^, ^6^, ^7^ However, a pathological link between DNM3OS and HD has not yet been reported.

In the present study, we explored the expression levels of DNM3OS, miR‐196b‐5p and GAPDH in HD PC12 cells. In addition, we identified a regulatory relationship among DNM3OS/miR‐196b‐5p/GAPDH and explained the involvement of this axis in mediating GAPDH‐induced pathological aggregate formation in HD.

## 2 MATERIALS AND METHODS

### 2.1 Cell lines and culture

PC12 cells induced by rat pheochromocytoma expressing HD gene exon 1 fragment with either 23 (httex1p‐Q23, normal; two independent cell lines) or 74 (httex1p‐Q74, mutant; two independent cell lines) polyglutamine repeats fused to the green fluorescent protein (GFP) were cultured and induced by doxycycline (dox) as described previously.^8^ The cells were cultured in 10% CO_2_ at 37°C in standard high‐glucose Dulbecco's modified Eagle medium (DMEM; Invitrogen Life Technologies) supplemented with 100 U/ml penicillin‐streptomycin (Invitrogen Life Technologies), 2 ml glutamine (Invitrogen Life Technologies), 5% Tet‐approved foetal bovine serum (Clontech), 100 Ag/ml G418 (Invitrogen Life Technologies) and 75 Ag/ml hygromycin (Invitrogen Life Technologies).

### 2.2 RNA extraction and quantitative real‑time PCR (RT‑qPCR)

Total RNA was extracted from httex1p‐Q74 cells using TRIzol reagent (Life Technologies Corporation, Carlsbad, CA). A Nanodrop™ spectrophotometer (ND‐100; Thermo, Waltham, MA) was used to determine the concentration and quality of RNA at 260/280 nm. RT‐qPCR detection of DNM3OS was performed using a Two‐Step SYBR^®^ Primer Script™ RT‐PCR Kit (Takara Bio, Inc., Japan). cDNA was synthesized using a TaqMan mRNA Reverse Transcription Kit (Applied Biosystems, Foster City, CA, USA). TaqMan Universal Master Mix II was implemented for miR‐196b‐5p and *U6* (Applied Biosystems) assays using the ABI 7500 Fast Real‐Time PCR System (Applied Biosystems). The fold change was calculated and the expression levels were normalized to endogenous controls using the relative quantitation (2^−ΔΔCt^) method. *U6* and *β*‐*actin* were used for the normalization of miRNA and lncRNA, respectively. The primer sequences were as follows: β‐actin, forward, 5′‐CTCCATCATGAAGTGTGACGTT‐3′, reverse, 5′‐ATCTCCTTCTGCATCCTGTCAG‐3′; U6, forward 5′‐GCTTCGGCAGCACATATACTAAAAT‐3′, reverse: 5′‐CGCTTCACGAATTTGCGTGTCAT‐3′; GAPDH, forward, 5′‐GGGAGCCAAAAGGGTCAT‐3′, reverse, 5′‐GAGTCCTTCCACGATACCAA‐3′; miR‐196b‐5p, forward, 5′‐ATCCTTCCTAGTCCAGCCTGAG‐3′; reverse, 5′‐ACCTGGCGGCACTCCTTA‐3′; DNM3OS, forward, 5′‐GGTCCTAAATTCATTGCCAGTTC‐3′ and reverse, 5′‐ACTCAAGGGCTGTGATTTCC‐3′.

### 2.3 Western blotting

Total protein was extracted from httex1p‐Q74 cells using ice‐cold RIPA buffer containing protease inhibitors (Sigma, St. Louis, MO, USA). An equal amount of each protein sample was separated by 12% SDS/PAGE and electrophoretically transferred to PVDF membrane. Subsequently, membranes were blocked in Tris‐buffered saline containing 5% non‐fat milk and 0.1% Tween 20 for 2 h at room temperature and incubated with the following primary antibodies at 4°C for at least 12 h: GAPDH (1:5000; Proteintech, Rosemont, IL) and β‐actin (1:5000; Proteintech, Rosemont, IL). The next day, membranes were incubated with secondary antibodies (goat anti‐rabbit or goat anti‐mouse, respectively, 1:4000; Proteintech Group, Chicago), and protein bands were visualized using an electrochemiluminescence detection system (ECL kit; Santa Cruz Biotechnology). Relative integrated density values (IDVs) were calculated using the ImageJ software with β‐actin as an internal control.

### 2.4 Cell transfection

To verify our hypothesis, we synthesized the following plasmids: short‐hairpin DNM3OS (sh‐DNM3OS) and its respective non‐targeting sequence (negative control; sh‐NC) (GenePharma, Shanghai, China). Cells were grown to 70%–80% confluence in 24‐well plates and transfected using Lipofectamine™ 3000 (Life Technologies Corporation, Carlsbad, CA) according to the manufacturer's instructions. Geneticin (G418; Sigma‐Aldrich, St. Louis, MO, USA) was used to select stably transfected cells. Three groups were designed to verify the effect of DNM3OS in httex1p‐Q74 cells: control, sh‐NC (transfected with empty plasmid) and sh‐DNM3OS.

miR‐196b‐5p mimics (mimic‐miR‐196b‐5p), miR‐196b‐5p inhibitors (inhibitor‐miR‐196b‐5p) and their respective non‐targeting sequences (mimic‐NC or inhibitor‐NC) were synthesized by GenePharma (Shanghai, China) and used to investigate the function of miR‐196b‐5p in httex1p‐Q74 cells. The httex1p‐Q74 cells were divided into five groups: control, mimic‐196b‐5p‐NC, mimic‐196b‐5p, inhibitor‐196b‐5p‐NC and inhibitor‐196b‐5p. To verify our hypothesis that DNM3OS functions by suppressing miR‐196b‐5p, httex1p‐Q74 cells were divided into five groups: control, sh‐DNM3OS‐NC + mimic‐miR‐196b‐5p‐NC, sh‐DNM3OS + mimic‐miR‐196b‐5p, sh‐DNM3OS‐NC + inhibitor‐miR‐196b‐5p‐NC and sh‐DNM3OS + inhibitor‐miR‐196b‐5p.

### 2.5 Cell viability assay

Cell viability was analysed using a CCK‐8 kit (Dojin, Japan). In brief, httex1p‐Q74 cells were seeded in 96‐well plates and incubated overnight. Subsequently, 10 μl CCK‐8 was added to each well and the plates were incubated at 37°C for 2 h. The absorbance value (OD) was measured at 450 nm using a microplate reader (BioTek, Winooski, VT, USA).

### 2.6 Apoptosis assay

Apoptosis was evaluated using the terminal‐deoxynucleotidyl transferase–mediated nick‐end labelling (TUNEL) assay and flow cytometry (FAS Calibur, BD). According to the manufacturer's instructions, httex1p‐Q74 cells were collected and incubated with Annexin V‐PE and 7‐aminoactinomycin D (7‐AAD) (Southern Biotech, Birmingham, AL) in binding buffer. Annexin V+/7‐AAD– cells were identified as early apoptotic, while Annexin V+/7‐AAD+ cells were identified as late apoptotic. Apoptosis was observed by TUNEL staining using an in situ cell death detection kit (Roche, Mannheim, Germany). The average number of TUNEL‐positive cells was calculated in five randomly selected regions.

### 2.7 ROS detection assay

DCFH‐DA was diluted 1:1000 in serum‐free medium to a final concentration of 10 µmol/L. Cells were resuspended in diluted DCFH‐DA to a concentration of 1–20 million/ml and incubated at 37℃ for 20 min; every 3–5 min, the tubes were inverted to ensure full contact of the probe with the cells. Subsequently, cells were washed three times with serum‐free medium to remove DCFH‐DA. Rosup was added as a positive control. The level of ROS was detected by flow cytometry.

### 2.8 Dual‐luciferase reporter assay

The putative binding sites of miR‐196b‐5p with DNM3OS and the GAPDH 3’‐UTR fragment were amplified by PCR and cloned into a pmirGLO Dual‐luciferase miRNA Target Expression Vector (Promega, Madison, WI, USA) to construct the luciferase reporter plasmids (DNM3OS‐wt and GAPDH‐wt) (GenePharma). The corresponding mutants (DNM3OS‐mut and GAPDH‐mut) (GenePharma) were synthesized by gene alteration of the putative binding sites. Httex1p‐Q74 cells were co‐transfected with wild‐type pmirGLO‐DNM3OS (or DNM3OS mutant) and miRNA plasmids using Lipofectamine™ 3000. The Dual‐Luciferase Reporter System (Promega) was used to evaluate luciferase activity 48 h post‐transfection. The 3′‐UTR sequence of GAPDH containing the putative miR‐196b‐5p binding sites and their mutant sequences were cloned into dual‐luciferase vectors. The procedures for transfection and measurement of luciferase activity were performed as described above.

### 2.9 Statistical analysis

The data are expressed as the mean ± SD of at least three independent experiments. Student's *t* test (two‐tailed) or one‐way/two‐way ANOVA was applied for comparison of more than two groups. The Mann‐Whitney U test was used to compare non‐normally distributed data. For correlation analysis of normal distribution data, Pearson correlation analysis should be used, while Spearman method should be used for correlation analysis of non‐normal distribution data. Data analyses were performed using the SPSS 21.0 software (SPSS, Chicago, IL); differences were considered statistically significant at *p* < 0.05.

## 3 RESULTS

### 3.1 Downregulated DNM3OS ameliorate biological behaviour of httex1p‐q74 cells

**FIGURE 1:**
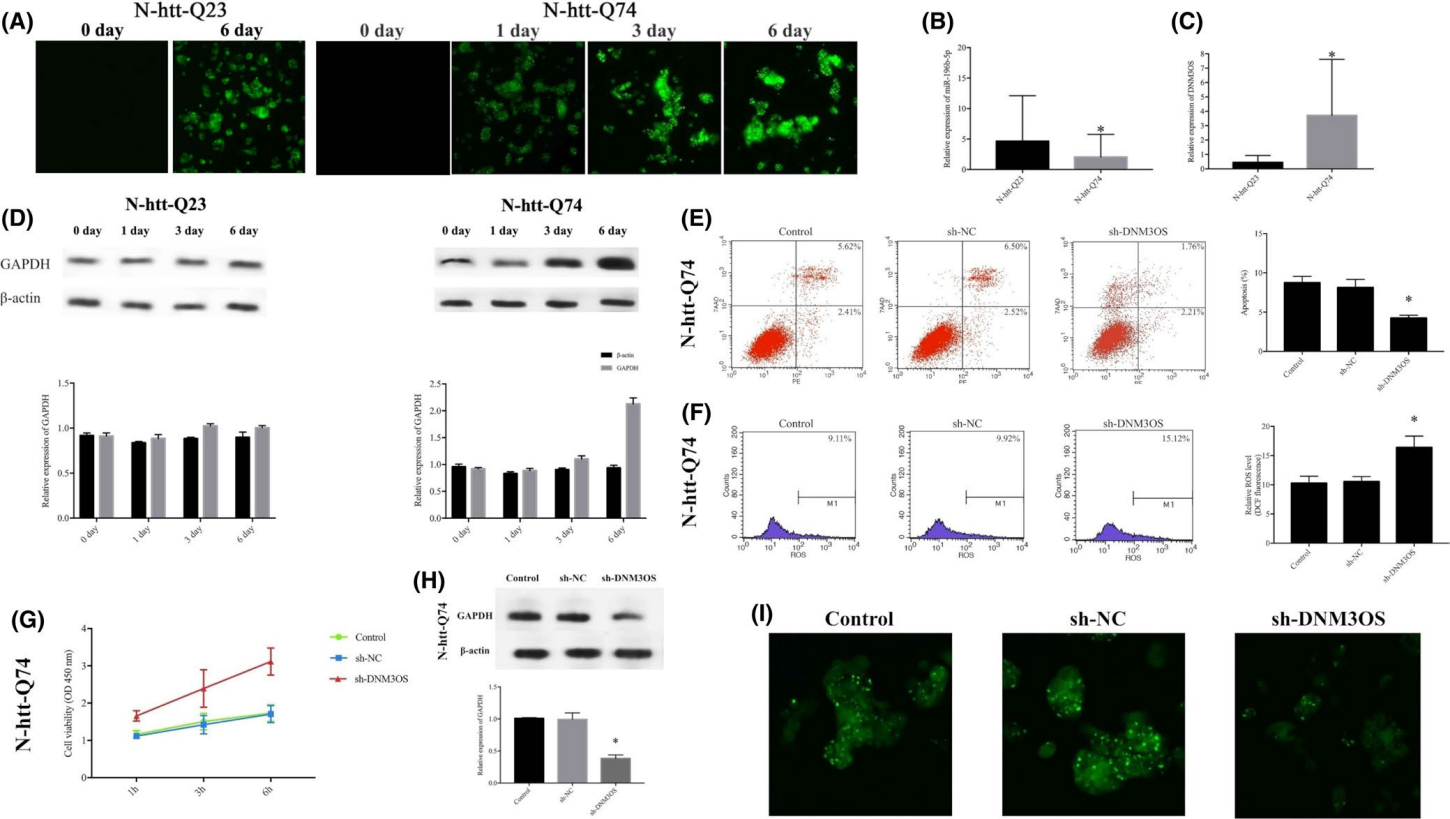
DNM3OS, miR‐196b‐5p and GAPDH expression in N‐htt‐Q23 and N‐htt‐Q74 cells. (A) HD PC12 cell line driven by the doxycycline‐dependent Tet‐On promoter to stably express the first exon fragment of *HTT* fused to GFP, containing either 23 (normal) or 74 (mutant) glutamine fragment (N‐htt‐Q23 or N‐htt‐Q74, respectively). (B) Expression levels of DNM3OS in N‐htt‐Q74 and N‐htt‐Q23 cells. (C) Expression levels of miR‐196b‐5p in N‐htt‐Q74 and N‐htt‐Q23 cells. Data represent the mean ± SD (*n* = 3, each group), *p < 0.05. (D) GAPDH protein expression levels in N‐htt‐Q23 and N‐htt‐Q74 cells relative to β‐actin as an endogenous control. Knockdown of DNM3OS affects the biological behaviour of N‐htt‐Q74 cells. (E) Flow cytometry analysis of DNM3O knockdown in N‐htt‐Q74 cells. Apoptosis rates equate to the sum of the percentages of the right lower (representing early apoptosis) and right upper (representing late apoptosis) quadrants. Statistical analysis was performed using a non‐parametric Mann‐Whitney test. *p < 0.05 vs. control group. (F) DCF fluorescence indicating the level of ROS in N‐htt‐Q74 cells was compared with the M1 Gated% using a non‐parametric Mann‐Whitney test. *p < 0.05 vs. control group. (G) A CCK‐8 assay was used to determine the effect of DNM3OS on N‐htt‐Q74 cell viability. (H) Western blotting of GAPDH protein expression in N‐htt‐Q74 cells following transfection with sh‐DNM3OS. β‐actin was used as an endogenous control. (I) Effect of DNM3OS inhibition on IB formation of the mHtt protein

In the present study, we used RT‐qPCR to evaluate the expression levels of DNM3OS and miR‐196b‐5p in HD PC12 cells. Figure 1A shows that PC12 cells driven by the doxycycline‐dependent Tet‐On promoter could be induced to stably express the first exon fragment of *HTT* fused to GFP, containing either 23 (normal) or 74 (mutant) glutamine fragment (httex1p‐Q23 or httex1p‐Q74 cells). The expression levels of miR‐196b‐5p, DNM3OS and GAPDH in httex1p‐Q74 cells were identified (Figure 1B‐D).

We established stable inhibition of DNM3OS (sh‐DNM3OS) in httex1p‐Q74 cells, ﻿and flow cytometry results show that inhibition of DNM3OS decreased the rate of apoptosis to lower than that of the sh‐NC group (Figure 1E). The amount of ROS in httex1p‐Q74 cells and levels in sh‐DNM3OS‐transfected httex1p‐Q74 cells significantly increased (Figure 1F). The viability of sh‐DNM3OS‐transfected httex1p‐Q74 cells was much higher, and there was an increasing trend over time (Figure 1G). The GAPDH was significantly downregulated in sh‐DNM3OS‐transfected httex1p‐Q74 cells (Figure 1H). Importantly, downregulation of DNM3OS reduced IB formation of the mHtt protein (Figure 1I), suggesting that inhibition of DNM3OS expression may have protective effects on HD PC12 cells.

### 3.2 Protective effects of miR‐196b‐5p in httex1p‐q74 cells and DNM3OS binds to and negatively regulates miR‐196b‐5p

**FIGURE 2:**
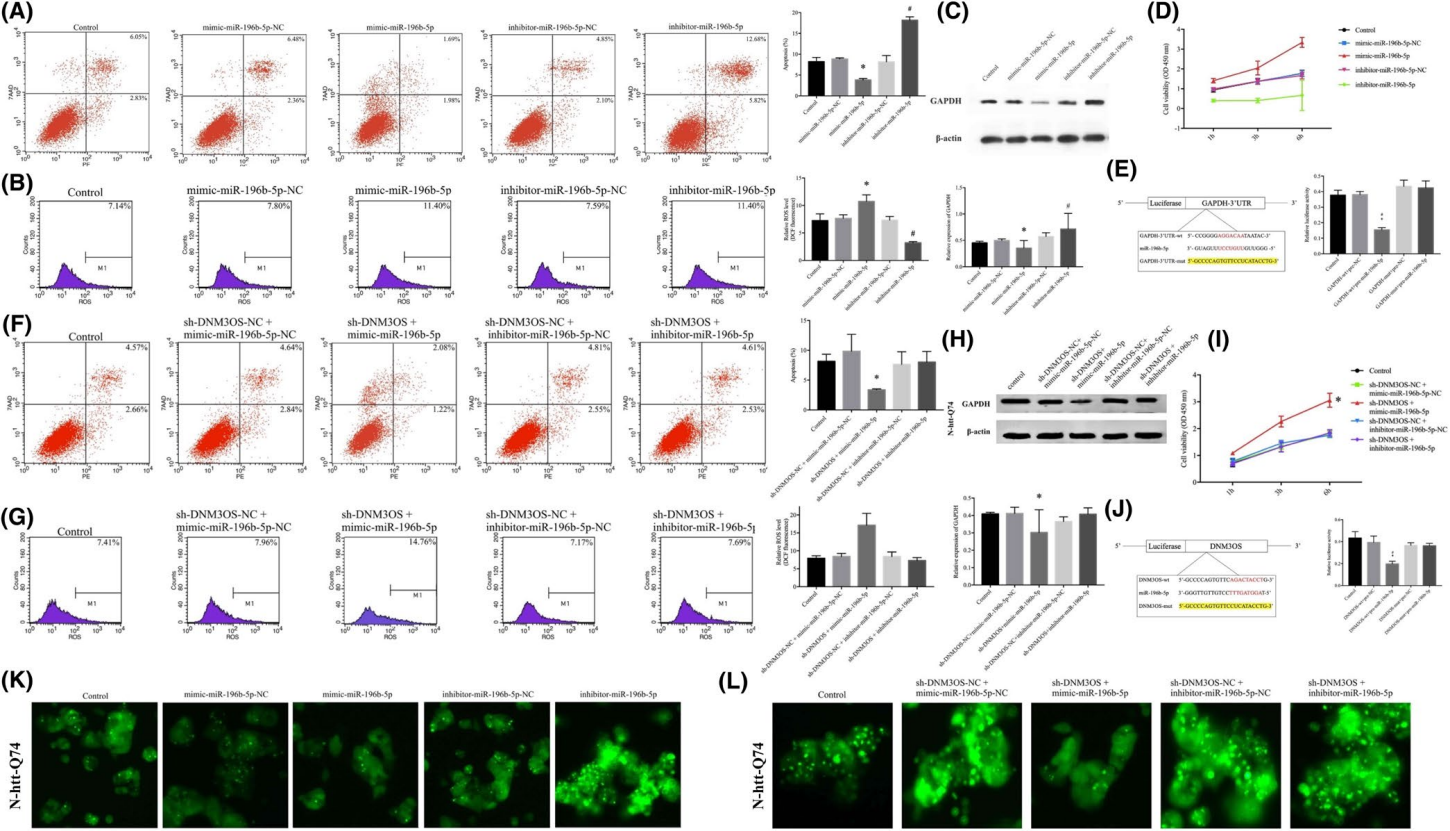
Effects of miR‐196b‐5p on the biological behaviour of N‐htt‐Q74 cells. (A) Flow cytometry analysis of n‐htt‐q74 cells following manipulation of mir‐196b‐5p expression. (B) Relative ROS levels in N‐htt‐Q74 cells as detected by DCF fluorescence following manipulation of miR‐196b‐5p expression. (C) The expression of GAPDH relative to β‐actin as an endogenous control. The IDVs of GAPDH following manipulation of miR‐196b‐5p relative to β‐actin as an endogenous control. *p < 0.05, ^#^ *p* < 0.05 vs. control group. (D) Dynamic changes in N‐htt‐Q74 cell viability in different groups following manipulation of miR‐196b‐5p. (E) Schematic of the putative binding site of miR‐196b‐5p with GAPDH (GAPDH‐wt) and the designed mutant sequence (GAPDH‐mut). The relative luciferase activities of N‐htt‐Q74 cells co‐transfected with GAPDH‐wt or GAPDH‐mut and pre‐miR‐196b‐5p or pre‐NC. *p < 0.05 vs. GAPDH‐wt + pre‐NC. ^#^ *p* < 0.05 vs. GAPDH‐mut + pre‐miR‐196b‐5p. (F) Flow cytometry analysis of N‐htt‐Q74 cells co‐transfected sh‐DNM3OS with inhibitor‐or mimic‐miR‐196b‐5. The apoptosis rate was significantly decreased in the sh‐DNM3OS + mimic‐miR‐196b‐5 group (*p < 0.05 vs. control group). (G) Relative ROS levels in N‐htt‐Q74 cells detected by DCF fluorescence following manipulation of DNM3OS and miR‐196b‐5 expression. (H) The expression of GAPDH relative to β‐actin as an endogenous control. The IDVs of GAPDH is following co‐transfection with sh‐DNM3OS and inhibitor or mimic‐miR‐196b‐5 relative to β‐actin as an endogenous control. *p < 0.05 vs. control group. (I) Dynamic changes in N‐htt‐Q74 cell viability in different groups following manipulation of DNM3OS and miR‐196b‐5 expression. (J) Schematic of the putative binding site of miR‐196b‐5p with DNM3OS (DNM3OS‐wt) and the designed mutant sequence (DNM3OS‐mut). The relative luciferase activities of N‐htt‐Q74 cells co‐transfected with DNM3OS‐wt or DNM3OS‐mut and pre‐miR‐196b‐5p or pre‐NC. *p < 0.05 vs. DNM3OS‐wt +pre‐NC. ^#^ *p* < 0.05 vs. DNM3OS‐mut +pre‐miR‐196b‐5p. k. Effects of manipulation of miR‐196b‐5p expression on IB formation in N‐htt‐Q74 cells. (L) Effects of manipulation of DNM3OS and miR‐196b‐5 expression on IB formation in N‐htt‐Q74 cells

We subsequently established stable overexpression or inhibition of miR‐196b‐5p in httex1p‐Q74 cells. The apoptosis rate was much lower in the miR‐196b‐5p overexpression group, while it was significantly higher in the inhibitor‐miR‐196b‐5p group (Figure 2A). The ROS level was significantly higher in the mimic‐miR‐196b‐5p group and lower in the inhibitor‐miR‐196b‐5p group (Figure 2B). Western blotting shows that GAPDH was scarcely expressed in the mimic‐miR‐196b‐5p group and highly expressed in the inhibitor‐miR‐196b‐5p group (Figure 2C). Moreover, there were obvious differences in cell viability among the different groups; the inhibitor‐miR‐196b‐5p group had the lowest cell viability, while the mimic‐miR‐196b‐5p group had the highest cell viability (*p* < 0.05; Figure 2D).

A bioinformatics database (TargetScan) provided a putative target binding site between miR‐196b‐5p and the GAPDH 3′‐UTR, and dual‐luciferase activity was measured to identify the 3′‐UTR of GAPDH as a direct downstream target of miR‐196b‐5p. Cells transfected with both GAPDH‐Wt and pre‐miR‐196b‐5p (GAPDH‐Wt + pre‐miR‐196b‐5p) showed significantly impaired luciferase activity as compared with the other groups (Figure 2E). As presented in Figure 2K, IBs were reduced in the mimic‐miR‐196b‐5p group but markedly increased in the inhibitor‐miR‐196b‐5p group, suggesting that overexpression of miR‐196b‐5p may have protective effects in HD PC12 cells.

Further, we co‐transfected sh‐DNM3OS with inhibitor or mimic‐miR‐196b‐5p to investigate the effects on the biological behaviour of httex1p‐Q74 cells. The apoptosis rate was significantly reduced in the sh‐DNM3OS + mimic‐miR‐196b‐5p group (Figure 2F), and the ROS levels and cell viability were markedly improved (Figure 2G). In addition, the expression level of GAPDH was decreased in the sh‐DNM3OS + mimic‐miR‐196b‐5p group (Figure 2H). The cell viability was significantly increased in the sh‐DNM3OS + mimic‐miR‐196b‐5p group as compared with the other groups (Figure 2I). According to StarBase (http://starbase.sysu.edu.cn), there may be a regulatory relationship between DNM3OS and miR‐196b‐5p. We performed a dual‐luciferase reporter assay to demonstrate that DNM3OS can bind to miR‐196b‐5p at the predicted binding sites. The luciferase activity was analysed (Figure 2J), and we found the DNM3OS can bind to miR‐196b‐5p and exert a negative regulatory effect. We also found that IBs of the mHtt protein were significantly reduced in the sh‐DNM3OS + mimic‐miR‐196b‐5p group (Figure 2L).

## 4 DISCUSSION

In the present study, we explored the role of DNM3OS in HD pathogenesis for the first time and investigated its effects on IB formation of the mHtt protein in httex1p‐Q74 cells by modulating GAPDH expression. Here, we identified that GAPDH can directly bind to its 3’‐UTR, and hence, DNM3OS knockdown could inhibit IB formation of the mHtt protein in httex1p‐Q74 cells through the DNM3OS/miR‐196b‐5p/GAPDH axis.

Previous studies have confirmed that the elongated poly Q stretch in mHtt correlates well with the pathology of HD, and inhibition of mHtt aggregation may be a promising therapeutic strategy to inhibit the progression of HD.^8^, ^9^ In the present study, httex1p‐Q74 cells transfected with sh‐DNM3OS showed a decrease in the apoptosis rate and an increase in the ROS level and cell viability as compared with the control groups. Additionally, we found that these changes may be due to the decrease in GAPDH expression induced by sh‐DNM3OS, which may affect the cytotoxicity of mHtt aggregates; however, the complex mechanism underlying the role of DNMOS in regulating cell viability needs to be further explored.

To investigate the molecular regulatory mechanism of DNM3OS in IB formation of the mHtt protein, we further detected the downstream target miRNAs of DNM3OS and found that miR‐196b‐5p was downregulated and may be a direct target of DNM3OS. In the present study, we demonstrated that miR‐196b‐5p was downregulated in httex1p‐Q74 cells, while DNM3OS was upregulated. Overexpression of miR‐196b‐5 improved the apoptosis rate, ROS levels and cell viability in httex1p‐Q74 cells; however, knockdown of miR‐196b‐5 produced the opposite effect following transfection of cells with sh‐DNM3OS. Consequently, our results reveal miR‐196b‐5 to be a possible competing endogenous RNA of DNM3OS that may regulate its expression and function.

According to the TargetScan database, miR‐196b‐5p may target the GAPDH 3′‐UTR. In HD, GAPDH was one of the first proteins identified to interact with mHtt.^10^ In addition, GAPDH is an important participant in the poly Q aggregation process and enhances its cytotoxicity.^11^ Previous research has confirmed a protective effect of GAPDH expression regulation in the HD cell model.^12^, ^13^ Overexpression of GAPDH enhances the nuclear translocation and cytotoxicity of mHtt, whereas depletion of GAPDH by RNA interference diminishes these characteristics.^14^, ^15^ Since GAPDH participates in IB formation of the mHtt protein and can bind to miR‐196b‐5p, we hypothesized that the expression of GAPDH is mediated by miR‐196b‐5p. In the present study, GAPDH levels were downregulated and IB formation of the mHtt protein was repressed in the mimic‐miR‐196b‐5p group, suggesting that miR‐196b‐5p may regulate this process via the GAPDH pathway. In the present study, we improved the apoptosis rate, ROS levels, cell viability and IB formation of the mHtt protein in httex1p‐Q74 cells through the modulation of DNM3OS by targeting miR‐196b‐5p and GAPDH.

In conclusion, our results reveal that DNM3OS is upregulated in httex1p‐Q74 cells and the DNM3OS/miR‐196b‐5p/GAPDH axis is involved in regulating biological behaviour and IB formation of the mHtt protein, which may offer a potential therapeutic strategy for HD.

## CONFLICT OF INTEREST

The authors declare that they have no conflict of interests.

## AUTHOR CONTRIBUTIONS

**Xiaoyu Dong:** Data curation (equal); Formal analysis (equal); Investigation (equal); Writing‐original draft (equal). **Shuyan Cong:** Conceptualization (lead); Funding acquisition (lead); Supervision (lead).

## Data Availability

The data sets analyzed during the current study are available from the corresponding author on reasonable request.
